# Enhanced Electrochemical Performance of Tin Oxide Quantum Dots on Reduced Graphene Oxide under Light

**DOI:** 10.3390/mi15091125

**Published:** 2024-09-02

**Authors:** Itheereddi Neelakanta Reddy, Bhargav Akkinepally, Jaesool Shim, Cheolho Bai

**Affiliations:** School of Mechanical Engineering, Yeungnam University, Gyeongsan 38541, Republic of Korea

**Keywords:** nanosheets, heterostructures, electrolyte: electrochemical properties, photocurrent density

## Abstract

The study utilized a simple and cost-effective approach to improve the photoelectrochemical (PEC) water-splitting performance of various materials, including reduced graphene oxide (rGO), tin oxide nanostructures (SnO_2_), and rGO/SnO_2_ composites. The composites examined were rS15, containing 15 mg of rGO and 45 mg of SnO_2_, and rS5, with 5 mg of rGO and 50 mg of SnO_2_, tested in a sodium hydroxide (NaOH) electrolyte. Notably, the rS5 electrode showed a significant increase in PEC efficiency in 0.1 M NaOH, achieving a peak photocurrent density of 13.24 mA cm^−2^ under illumination, which was seven times higher than that of pristine rGO nanostructures. This enhancement was attributed to the synergistic effects of the heterostructure, which reduced resistance and minimized charge recombination, thereby maximizing the catalytic activity across the various electrochemical applications. Furthermore, the rS5 anode demonstrated improved Tafel parameters, indicating faster reaction kinetics and lower overpotential for efficient current generation. These results highlight the potential for optimizing nanostructures to significantly enhance PEC performance, paving the way for advancements in sustainable water-splitting technologies.

## 1. Introduction

Global concerns regarding environmental pollution and the energy crisis have intensified recently. Several approaches have been employed to comprehend and enhance photocatalytic and photoelectrocatalytic activity and photoelectrochemical glycerol oxidation, including the use of Density-Functional Theory [1,2]. A promising solution is to generate clean energy through solar-driven photoelectrochemical (PEC) water splitting, which has garnered significant interest from researchers [3,4]. Non-metal oxide semiconductors, particularly those with tunable bandgaps, have been extensively studied and utilized as photoelectrodes for PEC water splitting. Their appeal stems from PEC stability, raw material abundance, and cost-effective synthesis methods [5,6,7].

Reduced graphene oxide (rGO) is derived from graphene oxide using modification techniques, such as chemical, electrochemical, photonic, microwave, hydrothermal, and solvothermal methods [8]. Among these, microwave reduction is particularly promising for producing tunable, scalable, and high-quality graphene-based nanomaterials [9]. Depending on the reduction method and synthesis conditions, rGO morphology can vary substantially, forming foams, sponges, hydrogels/aerogels, sheets, interconnected networks, and fibers. rGO is extensively used for its efficient electrochemical activity owing to its large surface area and high affinity for binding functional groups [10]. Recently, rGO-based electrocatalysts have garnered significant interest as electron conductors for water-based conversion reactions [11]. Additionally, rGO offers several advantages as a photoelectrode for PEC activity, such as a large surface area that provides ample active sites for reactions and tunable electronic properties achieved through various reduction methods that optimize light absorption and charge transport. Furthermore, rGO’s excellent conductivity facilitates efficient electron transfer and reduces recombination loss. Its chemical stability and ability to form composites with other semiconductors improve the durability and performance of PEC systems, and its cost-effective and scalable synthesis makes it practical for large-scale applications in solar-driven water splitting. However, its inherent structural and electronic properties limit its catalytic activity [12]. Primarily, defects and oxygen-containing functional groups act as charge carrier traps, hindering efficient charge transport and increasing electron-hole recombination rates, thereby reducing overall photocurrent efficiency. The reduction results in a material with a smaller surface area than pristine graphene, limiting the number of active sites available for photochemical reactions and directly affecting charge separation efficiency. In addition, the variability in the quality and consistency of rGO produced using different synthesis methods can lead to inconsistent performances. The modified band structure of rGO, influenced by residual oxygen groups, can create unfavorable energy-level alignments for effective charge transfer processes. Additionally, incorporating rGO into photoelectrodes often requires complex fabrication processes, posing challenges to scale-up industrial applications. Advanced strategies such as controlled functionalization, nanostructuring, doping, and creating hybrid composites have been employed to improve the electronic properties, surface area, and overall charge dynamics of rGO-based photoelectrodes.

Tin dioxide (SnO_2_) offers a wide bandgap and high electron mobility, facilitating the rapid transport of photogenerated electrons and minimizing recombination losses, enhancing overall efficiency. Its compatibility with doping and surface functionalization extends its versatility and catalytic activity, positioning it as a promising material for scalable and cost-effective applications in sustainable energy and environmental technologies. One effective strategy involves creating heterojunctions with complementary semiconductor materials, which enhances light response ability and promotes the efficient separation of electron–hole pairs. Laura et al. developed the heterostructure of a BiVO_4_/SnO_2_ photoanode, achieving a current density of 0.662 mAcm^−2^ [13]. Tuan et al. [14] developed SnO_2_/rGO nanocomposites for the catalytic degradation of methylene blue, achieving a 93.4% degradation under visible light. The photocatalytic and anticancer activities of the SnO_2_-rGO nanocomposite studied by Alaizeri et al. [15] achieved a 2-fold degradation of methylene blue and 1.5-fold enhanced activity against human liver cancer (HepG2) compared to the pristine sample. Karim et al. [16] studied the synergistic performance of a Fe_3_O_4_/SnO_2_/rGO nanocomposite for energy storage and catalytic activity, achieving a specific capacitance of 967.5 F g^−1^ and a 92% degradation of methylene blue. Fang et al. elaborated nano-heterostructures for conversion applications [17]. In this study, a novel rGO-SnO_2_ heterostructure was developed as a photoelectrode for PEC applications. The rGO-SnO_2_ heterostructure optimizes PEC activity through several mechanisms. First, the hetero-junction between the rGO and SnO_2_ facilitates efficient charge separation and migration by leveraging their synergistic electronic properties, which minimizes recombination losses and boosts photocurrent density. Second, the rGO serves as a conductive network that enhances electron transport within the photoelectrode, reducing the charge transfer resistance and enhancing the efficiency of PEC reactions. Third, the unique structural properties and large surface area of the rGO-SnO_2_ heterostructure provide ample active sites for catalytic reactions, such as water oxidation or reduction, which are crucial for generating hydrogen or oxygen in PEC cells. Overall, the design of the rGO-SnO_2_ heterostructure combines enhanced charge separation, improved electron transport, and increased catalytic activity, enhancing the overall performance of the photoelectrode in PEC applications.

## 2. Materials and Methods

### 2.1. Materials Synthesis 

Reduced graphene oxide (rGO) was efficiently synthesized via microwave irradiation. We placed 100 mg of graphene oxide (GO; Sigma-Aldrich, St. Louis, MO, USA) in a 10 mL airtight glass vial and exposed it to microwave irradiation for 80 s. This treatment effectively reduced the oxygen-containing functional groups on the GO, converting it to rGO.

Next, we dissolved 2.0 g of SnCl_4_·5H₂O (98%, Sigma-Aldrich, St. Louis, MO, USA) in 100 mL of water and stirred it for 90 min. Then, 5 mL of hydrazine (N_2_H_4_; 98%, Sigma-Aldrich, St. Louis, MO, USA) was added at 25 °C under constant stirring. The mixture was allowed to react at 115 °C for 19 h without stirring. After cooling, the product was purified and extracted by repeated centrifugation with water and ethanol at 8000 rpm and then dehydrated at 85 °C for 24 h in an oven.

Varying weights of the synthesized rGO and SnO_2_ nanostructures were separately dispersed in 40 mL of H_2_O using probe sonication for 2 h. SnO_2_ was then slowly added dropwise to a pre-sonicated rGO solution, followed by continued sonication for 3 h to promote interactions between the components. The resulting composite was isolated via centrifugation, thoroughly washed with water and ethanol at 8000 rpm, and, finally, dehydrated in an oven at 80 °C for 24 h. The samples were denoted as follows: rGO as r, SnO_2_ as S, 15 mg of rGO and 45 mg of SnO_2_ as rS15, and 5 mg of rGO and 50 mg of SnO_2_ as rS5.

Each synthesized sample (2 mg) was dispersed in 3 mL of ethylene glycol using a sonicator for 5 min to achieve uniform dispersion. The resulting mixture was drop-cast onto a pre-cleaned 1 × 1 cm^2^ ITO glass substrate placed on a hot plate at 130 °C. After drop-casting, the substrate was transferred to an oven and dried at 130 °C for 24 h to ensure complete solvent evaporation and stable coating.

### 2.2. Characterization Techniques

The synthesized materials were characterized using various techniques. X-ray diffraction (XRD) (PANalytical X’pert PRO, Almelo, The Netherlands) was used to investigate the crystalline phase. Sample morphologies were examined using scanning electron microscopy (Hitachi S-4800, Tokyo, Japan). X-ray photoelectron spectroscopy (XPS) (Thermo Fisher Scientific MultiLab 2000, Seoul, Republic of Korea) was used to study the chemical states and valence bands. The optical properties were assessed using Fourier transform infrared spectroscopy (FT-IR) (Perkin Elmer Spectrum 100, Shelton, CT, USA), Raman spectroscopy (XploRA Plus, Osaka, Japan), and UV–Vis spectroscopy (Neogen NEO-D3117, Sejong-si, Republic of Korea).

The PEC water-splitting experiments were conducted using a three-electrode setup with an SP-200 potentiostat (Bio-Logic, Seyssinet-Pariset, France). Standard reference and counter electrodes of Ag/AgCl and Pt, respectively, were utilized. The fabricated electrodes served as the working electrodes, and all parameters were recorded against the reference electrode in the 0.1 M NaOH electrolyte under ON and OFF states. Electro-chemical impedance analysis was performed at a potential of 10 mV over a frequency range of 0.5–100 MHz in the same electrolyte. All the samples were exposed to a light source from ABET Technologies, Inc. (Model 10500, Milford, CT, USA), with a power density of 100 mW/cm^2^ at a distance of 6 cm.

## 3. Results and Discussion

**Figure 1:**
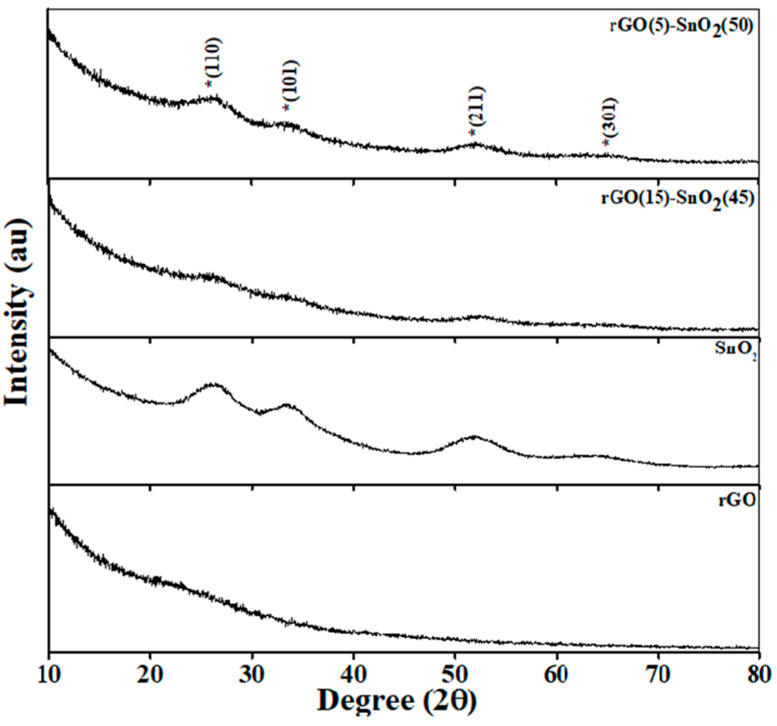
X-ray diffraction of the synthesized samples (* indicates the peaks of SnO_2_).

The phase analysis of the synthesized nanostructures is shown in Figure 1. The pristine rGO nanostructures exhibited amorphous structures. In contrast, the SnO_2_ nanostructures exhibited crystalline structures with characteristic peaks at 26.2° (110), 33.7° (101), 51.8° (211), and 64.6° (301) [18]. Furthermore, the prepared rS15 and rS5 hetero-structures exhibited pristine crystalline structures with no deviations in the peak positions. However, the rS15 samples showed low-intensity SnO_2_ characteristic peaks, possibly due to the rGO-layered structures covering the SnO_2_ nanostructure surfaces, suppressing the direct focus of the X-rays on the SnO_2_ surfaces. There were no variations in the intensities of the SnO_2_ peaks in the rS5 sample, likely owing to the low rGO content during heterostructure formation.

**Figure 2:**
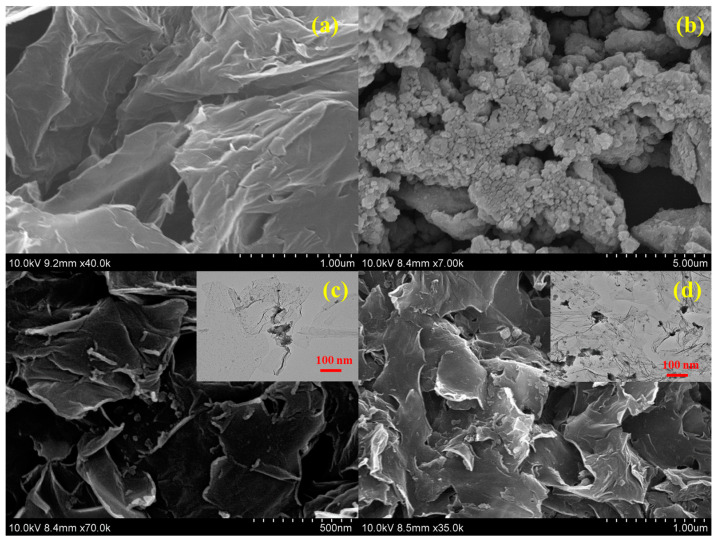
SEM images of the synthesized samples. (**a**) rGO, (**b**) SnO_2,_ (**c**,**d**) rGO-SnO_2_ composition samples.

The morphologies of the synthesized samples are shown in Figure 2. The rGO samples shown in Figure 2a featured wrinkled nanosheets with folded edges [19], whereas the SnO_2_ sample shown in Figure 2b consisted of clustered nanoparticles. In the rS15 heterostructure sample, the SnO_2_ nanoparticles were spread across the rGO nanosheets without altering the rGO morphology, as shown in Figure 2c. Finally, Figure 2d shows that the rS5 sample combined the original rGO and SnO_2_ forms, with a sparse distribution of SnO_2_ on the rGO surface owing to its low initial SnO_2_ content.

**Figure 3:**
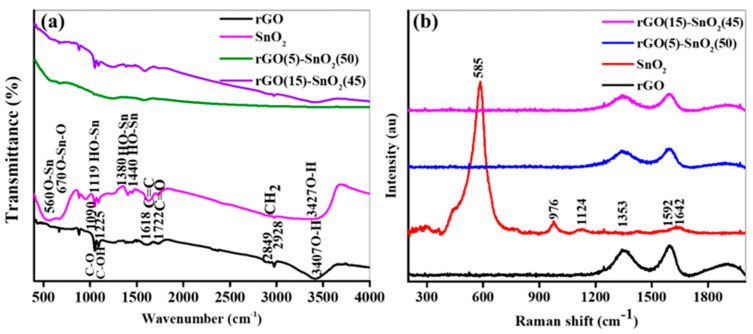
FTIR(**a**) and Raman (**b**) graphs of the synthesized samples.

FT-IR spectroscopy is an effective method for identifying various functional groups in synthesized samples. The FT-IR spectra of the rGO, SnO_2_, rS5, and rS15 samples are shown in Figure 3a. The rGO sample exhibited functional groups such as O–H, C–OH, carboxyl (COOH), and C–O [20,21,22]. A broad peak between 3500 and 2500 cm^−1^ in the rGO sample could be attributed to the carboxyl O–H stretching mode. The absorption peak at approximately 3407 cm^−1^, overlapping with the O–H stretch of carboxylic acid, indicated absorbed water molecules and alcohol groups. The characteristic peaks at 2928 and 2849 cm^−1^ were due to the asymmetric and symmetric CH_2_ stretching of the rGO, respectively, while the peak at approximately 1618 cm^−1^ corresponded to conjugated double bond (C=C) stretches from the graphitic domain. The peaks at approximately 1722, 1225, and 1090 cm^−1^ could be attributed to the carbonyl (C=O) stretch of the carboxyl group, C–OH stretch of the alcohol group, and C–O stretching vibrations of C–O–C, respectively. The SnO_2_ sample displayed absorption bands at 3472, 2919, 2880, 1440, 1380, 1119, 670, and 560 cm^−1^. The band at 3472 cm^−1^ could be attributed to O–H stretching vibrations and water molecules absorbed from the atmosphere on the SnO_2_ surface. The bands at 2919 and 2880 cm^−1^ indicated C–H groups. The bands at 1440, 1380, and 1119 cm^−1^ were associated with Sn-OH vibrations. The bands at 670 and 560 cm^−1^ corresponded to the stretching and vibrations of the O–Sn–O and Sn–O, respectively. Additionally, the heterostructure exhibited combined bands of rGO and SnO_2_ nanostructures, with no extra peaks observed, suggesting the high quality of the heterostructure. However, the rS5 sample exhibited low-intensity characteristic IR peaks, possibly owing to the SnO_2_ nanostructures enveloping the rGO surface, preventing the beam from directly focusing on the rGO architecture.

The Raman spectra of the four samples are shown in Figure 3b. In the rGO spectrum, characteristic G and D bands [23], typical of carbon nanostructures, were observed. The G-band, attributed to the C−C stretching in the carbon materials, was observed at 1592 cm^−1^. The D-band, associated with a disorder caused by oxygen moieties, appeared at 1353 cm^−1^ and was caused by the breathing mode of the aromatic rings. In addition, the Raman spectra revealed prominent bands at 585, 976, 1124, and 1642 cm^−1^. The band at 585 cm^−1^ corresponded to the Eg mode, indicating the symmetric stretching vibrations of the oxygen atoms bonded to the Sn in the SnO_2_ lattice. The second-order overtone at 976 cm^−1^ reflected the 2Eg mode, highlighting the high-energy vibrations of the O–Sn–O bonds. The band observed at 1124 cm^−1^ could be attributed to the A1g mode and was indicative of asymmetric stretching vibrations around the Sn atoms. Additionally, the band at 1642 cm^−1^ involved coupling the Eg mode and oxygen breathing vibrations, providing insights into the lattice dynamics and phonon interactions within the SnO_2_. Furthermore, the heterostructure displayed bands characteristic of rGO nanostructures without observable peaks of the SnO_2_. The absence of SnO_2_ peaks suggested that the SnO_2_ nanoparticles were intricately interlocked within the rGO nanosheets of the heterostructure.

**Figure 4:**
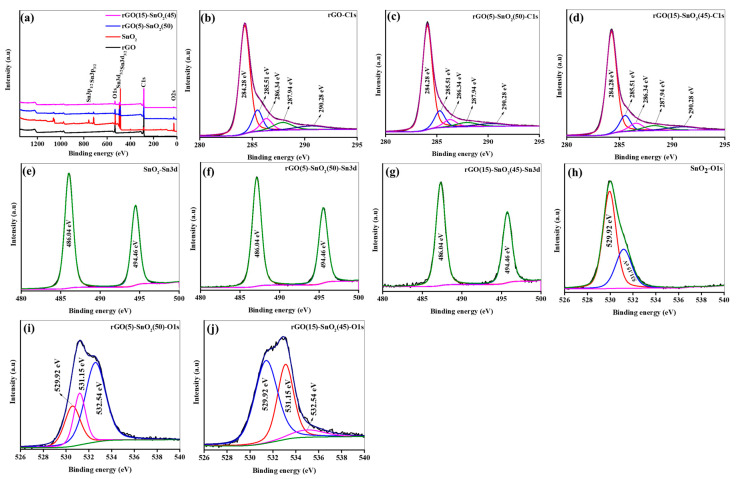
XPS analysis of the synthesized samples. (**a**) Survey (**b**–**d**) C1s of prepared samples, (**e**–**g**) Sn3d of the prepared samples (**h**–**j**) O1s of the prepared samples.

XPS was used to investigate the surface chemistry of the synthesized samples. The survey spectra of the samples shown in Figure 4a revealed distinct peaks corresponding to C1s (285.17 eV), Sn3d (492.09 and 486.46 eV), and O1s (528.55 eV) binding energies, and they indicated the purity of the synthesized samples. The deconvoluted XPS spectra of C1s, Sn3d, and O1s are shown in Figure 4b–j. Figure 2b–d show the high-resolution XPS spectra of C1s for the rGO, rS5, and rS15 samples. In all samples, the intensities of the C-C component substantially surpassed those of the oxygen groups (C=O and COOH), suggesting the existence of rGO. The C1s peaks of the rGO sample exhibited peaks at 284.28, 285.51, 286.34, 287.94, and 290.75 eV, which was indicative of various carbon chemical environments on the sample. The peak at 284.28 eV corresponded to carbon atoms in the sp2 hybridized C=C bonds [24]. This peak was characteristic of graphitic carbon being present in the rGO structure, indicating the presence of aromatic rings and double bonds between the carbon atoms within the graphene lattice. The peak at 285.51 eV was characteristic of carbon atoms bonded to oxygen atoms in various oxygen-containing functional groups. In rGO, this peak is often associated with C–O bonds, including epoxy (C–O–C), hydroxyl (C-OH), and C=O groups. These functional groups were remnants from the oxidation process of the graphene oxide and were partially retained after the reduction to rGO. Therefore, the C1s peak at 285.51 eV provided information about the oxygen-containing surface functional groups on the rGO material. The peak at 286.34 eV represented carbon atoms bonded to COOH. The COOH was represented by C=O and O–H bonds and was present in the rGO owing to the oxidation process involved in its synthesis. The C1s peak at 287.94 eV in the rGO corresponded to the carbon atoms bonded to the π-π* shake-up satellites or to the carbon atoms in the C=C within the graphene structure. These shake-up satellites were observed in the XPS spectra of the carbon-based materials and indicated excitations of the π electrons within the graphene lattice. Additionally, this energy range could include contributions from carbon atoms in the conjugated systems or those that bonded to oxygen in the specific functional groups. Finally, the C1s peak at 290.75 eV in the rGO corresponded to carbon atoms bonded to the carbonate groups (C–O–C) or other carbon–oxygen bonds associated with carbonates. Carbonates can form during the synthesis or processing of rGO, particularly if the material has undergone treatments involving carbon dioxide or carbonates as part of its functionalization process. The Sn3d peaks at 486.04 and 494.46 eV in all samples corresponded to distinct oxidation states of the Sn. The peak at 486.04 eV signified Sn in the +4 oxidation state (Sn^4+^), which is prevalent in SnO_2_, where Sn is tetrahedrally coordinated to the oxygen atoms in the rutile crystal structure. Conversely, the peak at 494.46 eV indicated Sn in a lower oxidation state, which is commonly associated with Sn^2+^. This suggested a reduced Sn state within the materials, which was potentially influenced by the synthesis conditions or surface interactions. In all samples, the O1s peaks observed at 529.92, 531.15, and 532.54 eV denoted distinct oxygen environments within these materials. The peak at 529.92 eV signified oxygen atoms bonded to metal ions, such as those in metal oxides (for, e.g., SnO_2_). The peak at 531.15 eV was associated with oxygen-containing functional groups such as C=O or the COOH groups present in the rGO, rS5, and rS15 samples. The peak at 532.54 eV indicated oxygen atoms bonded to organic contaminants such as adventitious carbonates or absorbed water molecules on the material surface.

**Figure 5:**
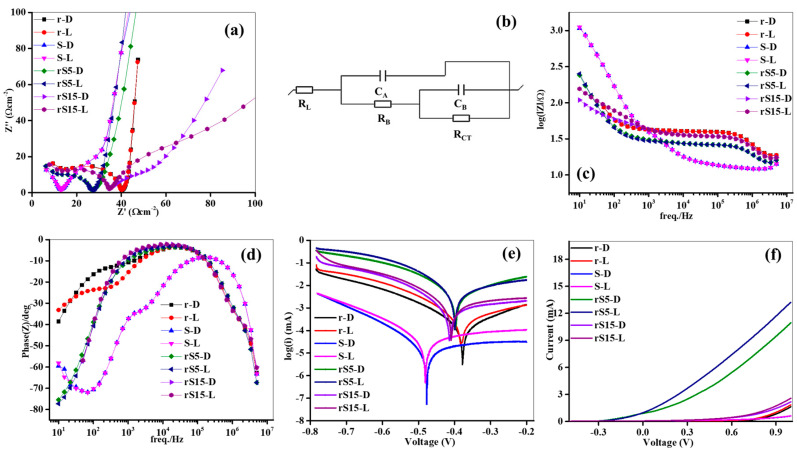
EIS, Bode, Phase, Tafel, and sweep voltammetry analyses of the synthesized samples. (**a**) Nyquist plots, (**b**) Fitted physical circuit (**c**) Bode plots (**d**) Phase plots (**e**) Tafel plots and (**f**) Linear voltammetry.

The charge kinetics of the four synthesized samples were examined using impedance spectroscopy in 0.1 M NaOH under dark (D) and light (L) states, as depicted in Figure 5a–d. The recorded Nyquist plots for the prepared samples in both states are shown in Figure 5a, displaying a half-circle at the low frequencies and a line at the mid-frequencies. The semicircular radius shown at the low frequencies indicated the carrier kinetics, whereas the mid-frequency line represented ion diffusion. The differences in the charge kinetics between the two states suggested that the samples effectively responded to the incident photons, generating carrier pairs under illumination. The Nyquist plots were analyzed using physical circuits to extract the kinetic variables, including the supporting solution resistance due to solid surface–liquid interactions (RL), internal resistance from the electrode boundary defects (RB), resistance associated with kinetics (RCT), bulk electrode capacitance (CA), and double-layer capacitance (CB), as shown in Figure 5b. The fitted variables for the r, S, rS15, and rS5 anodes are listed in Table 1. The anode resistance was lower in the light state than it was in the dark state, indicating efficient carrier transport to the electrode–solution interface. Under illumination, the rS5 anode exhibited a considerable RL value, indicating enhanced carrier movement across the anode surface–NaOH interface and improved electrochemical properties. The reduced RL value (2.33 Ω) for the rS5 anode compared to the pristine anodes could be attributed to the formation of heterostructures, which reduced the interface resistance, as noted in [25]. The rS5 anode had the lowest RB value (18.61 Ω) in the L state compared to the pristine nanostructures, suggesting that the semiconductor binding effectively reduced the carrier recombination rates due to variations in the optical band structures. This is crucial for achieving enhanced electrochemical performance. The recorded RCT of 0.49 kΩ for the rS5 anode under illumination was considerably lower than those for r (5.61 kΩ), S (1.82 kΩ), and rS15 (1.25 kΩ) under the same conditions. This indicated superior carrier migration towards the anode surface–solution interface in the rS5 anode, especially when the nanostructures were combined in the catalyst, enhancing the electrochemical activity under the L conditions. The CA and CB of the rS5 anode were higher than those of the other anodes in both states. The high CA and CB values (3.91 and 58.55 μF, respectively) obtained for the rS5 anode in the L state could be attributed to the heterostructures that prevented charge accumulation at the junction. These results suggested that the rS5 anode generated a superior current in the L state. Furthermore, the Bode and phase plots were crucial for evaluating the electron transport and recombination dynamics of the electrodes under the D/L conditions. The plots shown in Figure 5c,d provide detailed insights into the behavior of the samples in the NaOH electrolyte. The rS5 sample consistently exhibited lower impedance values at key frequencies in the NaOH electrolyte than the other samples, indicating superior charge transport efficiency. The low impedance facilitated easy charge movement, particularly under light exposure, with minimal phase angles. These minimal phase angles indicated efficient electron transport and a reduced likelihood of charge recombination, which were crucial for maximizing the sample’s PEC performance. In contrast, the other samples exhibited higher impedances and larger phase angles, indicating slower electron transport and higher recombination rates. These findings underscored the advantages of rS5 electrodes for effective charge transfer across different electrolytic environments, highlighting their potential to enhance PEC efficiency.

The Tafel plots depicted in Figure 5e offer a detailed examination of the electro-chemical characteristics of the r, S, rS15, and rS5 anodes, highlighting their behaviors under the L/D conditions. A notable positive shift in the potential was observed in the L state compared to that in the D state, indicating enhanced charge generation and catalytic activity under light exposure. Moreover, the rS5 anode exhibited a 30 mV potential shift in the L state compared to that of the pristine rGO, suggesting superior carrier generation facilitated by its heterostructures. Table 2 summarizes the key parameters from the Tafel experiments, including the Tafel slopes, limiting current density (JM), and exchange current density (JN), revealing lower slopes in the L state and indicating more efficient charge transfer kinetics. The rS5 anode demonstrated a Tafel slope of 48.3 mV dec^−2^ in the L state, reflecting faster charge transfer dynamics compared to those of the pristine anodes. Moreover, it achieved higher JM and JN values of −0.28 and −2.56 mA cm^−2^, respectively, in the L state, underscoring its enhanced carrier migration and catalytic activity under light conditions. These findings underscored the potential of rS5 nanostructures to enhance electrochemical performance driven by their structural advantages and efficient charge dynamics, making them promising candidates for advanced applications in energy conversion technology.

The linear sweep voltammetry data shown in Figure 5f provide detailed insights into the electrochemical behavior of the r, S, rS15, and rS5 anodes across a voltage range of −0.5 to +1.0 V in the 0.1 M NaOH electrolyte. As the applied voltage increased, all anodes showed progressive increases in current generation, reflecting their effective catalytic activity and charge transfer capabilities. Under illumination, each anode outperformed its D state, demonstrating responsiveness to light-induced effects. Among the anodes, rS5 consistently exhibited higher current densities due to the excellent conductivity of the rGO, which facilitated electron transfer, while SnO_2_ acted as a photoactive material, generating electron–hole pairs under illumination. The rGO content also enhanced charge separation, minimized recombination, and affected the dispersion of the SnO_2_, optimizing the surface area and the availability of active sites for the PEC reactions. Additionally, the rS5 anode’s performance was boosted by an optimized exchange interface that promoted efficient electron transport and minimized resistance losses, thereby improving electrochemical efficiency [26]. The currents generated by the developed photoanode were compared with published data, as shown in Table 3. The rS5 anode also demonstrated superior Tafel parameters, indicating faster reaction kinetics and a lower overpotential for current generation, achieving a peak current density of 13.24 mA cm^−2^ in the illuminated state, which was seven times higher than that of the r anode. This enhanced performance was due to the advanced heterostructures that reduced resistive losses and charge recombination, maximizing catalytic activity and ensuring robust performances across the diverse electrochemical applications. Overall, the exceptional performance of the rS5 anode highlights its potential as a highly efficient material for sustainable energy conversion technologies. 

**Figure 6:**
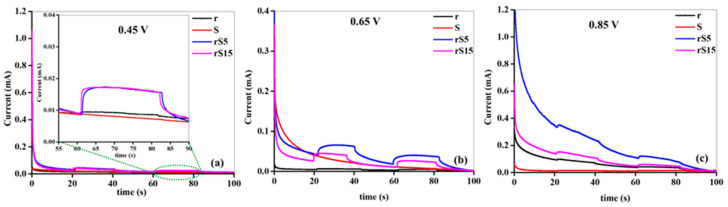
I-t analysis of the synthesized samples. (**a**) 0.45 V, (**b**) 0.65 and (**c**) 0.85 V.

**Figure 7:**
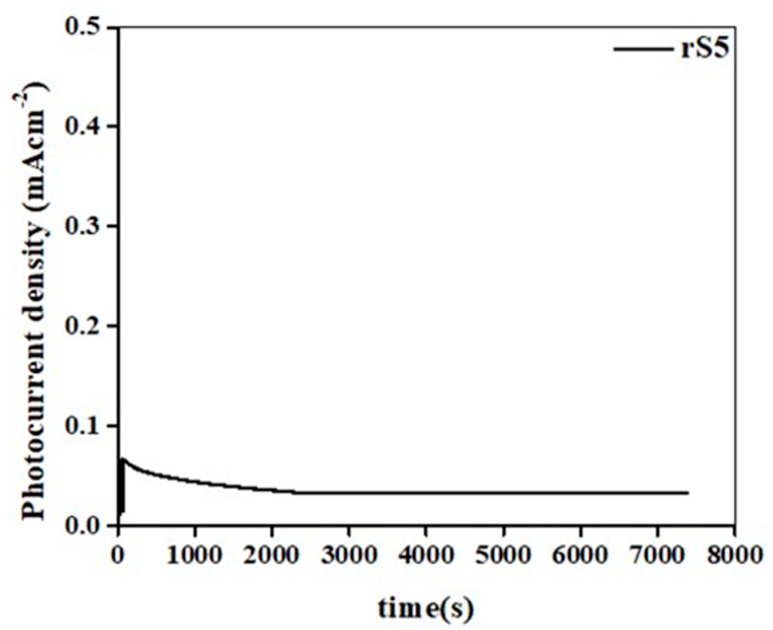
Long-term stability of the rS5 sample under extended illumination times.

The time-dependent evaluation of the developed structures depicted in Figure 6a–c underscored the critical influence of the applied voltage on the current output of the anodes. At 0.45 V, all anodes initially exhibited poor electrochemical performances in the L and D states; however, the heterostructure samples demonstrated distinct switching behaviors compared to their pristine counterparts, indicating heightened sensitivity to light-induced changes. Moreover, at 0.65 V, all anodes showed improved current generation, with the rS5 samples notably achieving the highest currents, surpassing the other configurations. This highlighted the superior capability of rS5 in facilitating electrochemical reactions under these conditions. Beyond 0.65 V, declines in current generation were observed across all anodes in the L and D states, suggesting an optimal voltage range for maximizing electrochemical activity. Notably, the currents were consistently higher in the L state than in the D state, underscoring the enhanced performance under light. The exceptional performance of the rS5 anode at these voltages could be attributed to the following factors: the development of effective heterostructures facilitating improved charge separation, reduced internal resistance, efficient electron transport, and an enhanced number of active sites promoting catalytic activity, optimized charge kinetics minimizing recombination losses, and increased capacitance that enhanced charge storage capacity. These factors collectively enhanced the overall electrochemical performance of the rS5 anode, emphasizing the significance of the applied potential in modulating carrier production and ensuring robust anode stability over extended operational periods. Furthermore, the long-term stability of the rS5 sample was evaluated, as shown in Figure 7. The rS5 sample demonstrated exceptional stability for up to 7300 s, with only a slight decrease in the generated current.

## 4. Conclusions

In this study, we successfully fabricated r, S, rS15, and rS5 electrodes to explore the effects of the heterostructure formations on their electrochemical properties. XRD and Raman spectroscopy confirmed the high quality of the rGO-SnO_2_ heterostructures. The rS5 heterostructure achieved a remarkable photocurrent density of 13.24 mA cm^−2^ at 1.0 V vs. Ag/AgCl, which was approximately eight times higher than that of the pristine rGO. This significant improvement is attributed to the inherent electric field generated by the heterostructure, promoting the migration of energized carriers, reducing the recombination rates, and enhancing the water-splitting efficiency. The strongly interconnected morphology of rS5 further improved the charge separation and water-splitting performance. Additionally, the rS5 samples exhibited increased charge densities and lower carrier transfer resistances at the anode–liquid interfaces, boosting current density. Thus, developing rGO-SnO_2_ heterostructures with robust interfacial connections is highly effective for enhancing the photo response of rGO anodes in water-splitting applications.

## Figures and Tables

**Table 1:** Impedance fitted values of the catalysts under dark and light states in the 0.1 M NaOH electrolyte.

Anode	State	R_L_ (Ω)	R_B_ (Ω)	R_CT_ (kΩ)	C_A_ (nF)	C_B_ (µF)
rGO (r)	Dark	9.55	30.02	8.51	7.01	95.85
	Light	4.69	28.63	5.61	3.88	85.41
SnO_2_ (S)	Dark	12.24	31.96	1.96	1.82	8.56
	Light	12.23	30.02	1.82	1.80	8.53
rGO(5)-SnO_2_(50) (rS5)	Dark	2.60	23.75	0.51	3.88	41.82
	Light	2.33	18.61	0.49	3.75	41.11
rGO(15)-SnO_2_(45)	Dark	6.48	25.02	1.95	4.01	59.77
(rS15)	Light	6.41	24.91	1.25	3.91	58.55

**Table 2:** Tafel fitted parameters of the synthesized samples.

Anode	State	Tafel Slope (mV dec^−1^)	J_M_ (mA cm^−2^)	J_N_ (mA cm^−2^)
rGO (r)	Dark	86.8	−1.27	−4.04
	Light	78.1	−1.09	−3.78
SnO_2_ (S)	Dark	66.4	−1.20	−5.11
	Light	60.3	−1.17	−4.75
rGO(5)-SnO_2_(50)	Dark	55.6	−0.34	−2.76
(rS5)	Light	48.3	−0.28	−2.56
rGO(15)-SnO_2_(45)	Dark	65.8	−0.78	−3.34
(rS15)	Light	52.2	−0.44	−3.17

**Table 3:** Comparison of the achieved photocurrents with published data.

c	Electrolyte	Current Density (mA cm^−2^)	References
rGO	0.5 M Na_2_SO_4_	2.5 × 10^−3^	[12]
SnO_2_	1 M H_3_BO_3_/KOH	0.3	[27]
BiVO_4_/rGO	Fe_S_O_4_·6H_2_O and Ni(NO_3_)_2_·6H_2_O	1.13	[28]
Bi_2_S_3_@rGO	0.1 M Na_2_SO_3_ and 0.1 M Na_2_S	6.06	[7]
rGO/MgFe_2_O_4_	1 M NaOH	0.15	[5]
ZnO:Ag/rGO	0.5MNa_2_SO_4_	0.2	[29]
rGO-SnO_2_	0.1 M NaOH	13.24	Present work

## Data Availability

The data are contained within the article.
